# Regulation of *C. elegans* Neuronal Differentiation by the ZEB-Family Factor ZAG-1 and the NK-2 Homeodomain Factor CEH-28

**DOI:** 10.1371/journal.pone.0113893

**Published:** 2014-12-04

**Authors:** Kalpana Ramakrishnan, Peter G. Okkema

**Affiliations:** Department of Biological Sciences, University of Illinois at Chicago, Chicago, Illinois, United States of America; Inserm U869, France

## Abstract

The *C. elegans* pharyngeal neuron M4 is a multi-functional cell that acts as a cholinergic motor neuron to stimulate peristaltic pharyngeal muscle contraction and as a neuroendocrine cell secreting neuropeptides and growth factors to affect other cells both inside and outside the pharynx. The conserved transcription factors ZAG-1 and CEH-28 are co-expressed in M4 through most of development, and here we examine how these factors contribute to M4 differentiation. We find ZAG-1 functions upstream of CEH-28 in a branched pathway to activate expression of different sets of M4 differentiation markers. CEH-28 activates expression of the growth factor genes *dbl-1* and *egl-17*, and the neuropeptide genes *flp-5* and *flp-2*, while ZAG-1 activates expression of the serotonin receptor *ser-7*, as well as expression of *ceh-28* and its downstream targets. Other markers of M4 differentiation are expressed normally in both *zag-1* and *ceh-28* mutants, including the neuropeptide gene *flp-21* and the acetylcholine biosynthetic gene *unc-17*. Unlike *ceh-28* mutants, *zag-1* mutants completely lack peristaltic muscle contractions resulting from broader defects in M4 differentiation. Despite these defects, neither ZAG-1 nor CEH-28 are terminal selectors of the M4 phenotype, and we suggest they function in a hierarchy to regulate different aspects of M4 differentiation.

## Introduction

Determining the mechanisms controlling motor neuron differentiation is essential to understanding nervous system development and to ultimately design cell-based therapies for human motor neuron diseases [reviewed in [1]]. However, the complexity of most nervous systems make it difficult to characterize these mechanisms for individual cell types.

The *C. elegans* pharynx is emerging as an exceptionally simple model to examine neuronal differentiation and function [2]. The pharynx is a rhythmically contracting neuromuscular pump located at the anterior of the digestive system, and it transports food through a central lumen into the intestine. The pharynx contains 20 neurons of 14 different types that make up a small nervous system separate from the somatic nervous system, and 20 muscle cells that contract during feeding [3]. These muscles exhibit two distinct types of contractions, called pumps and peristalses [4]. Pumping is a simultaneous contraction of the muscles in the anterior and very posterior regions of the pharynx, and these contractions concentrate food in the anterior pharyngeal lumen. In contrast, peristalsis is a wave-like contraction of a single muscle cell type that makes up a narrow region in the center of the pharynx called the isthmus, and this peristalsis carries a bolus of food through the isthmus lumen toward the intestine. Pumping occurs frequently, approximately 100–200 times per minute, while peristalses are relatively infrequent, occurring after every 4th to 40th pump. Our current challenge is understanding the mechanisms that produce the diverse neuron types that control pharyngeal contractions.

The pharyngeal M4 neuron is a multi-functional cell that both controls muscle contraction and secretes signaling molecules. M4 is a cholinergic motor neuron that stimulates isthmus muscle peristalsis, and in its absence the pharyngeal lumen becomes stuffed with food and the animals starve [5], [6]. Recently M4 has also been shown to have neurosecretory functions. M4 secretes the FMRFamide-like peptide neurotransmitter FLP-21 and the insulin-like growth factor INS-10, which function under hypoxic conditions to systemically modulate gustatory behavior and anterior touch neuron sensitivity, respectively [7], [8]. M4 also secretes the TGF-ß-family growth factor DBL-1 to affect the morphology of the nearby pharyngeal gland cells [9]. A number of additional neuropeptide and growth factor genes are also expressed in M4 [10], [11], and M4 can be considered part of a primitive neuroendocrine system [7], [9]. We are interested in how M4 differentiation is controlled to produce this complex, multifunctional phenotype.

The NK-2 family homeodomain transcription factor CEH-28 plays a key role in regulating synapse formation and gene expression in M4. *ceh-28* mutants exhibit abnormal and mispositioned synapses in M4 and a highly penetrant stuffed pharynx phenotype [12]. In contrast to animals that lack M4 and do not peristalse, *ceh-28* mutants can hyperstimulate isthmus muscle peristalses, and we believe this defect leads to inefficient feeding [5], [12]. *ceh-28* mutants fail to express the *dbl-1* gene in M4, and this loss of TGF-ß signaling leads to defects in morphology of the nearby g1 gland cells [9]. However other differentiation markers such as the serotonin receptor gene *ser-7b* and the vesicular ACh transporter gene *unc-17* are expressed normally in the M4 cell of *ceh-28* mutants [12]. Thus, other factors also contribute to M4 differentiation.

We are also interested in the role the conserved zinc-finger/homeodomain transcription factor ZAG-1 plays in M4. ZAG-1 is the sole *C. elegans* member of the ZEB-family of transcription factors, which in humans are mutated in Mowat-Wilson Syndrome and overexpressed in some metastatic cancers [reviewed in [13]]. *C. elegans zag-1* is widely expressed in the nervous system, including in M4, as well as in embryonic pharyngeal muscles [14], [15]. *zag-1* null mutants exhibit larval lethality and an inability to feed, and this feeding defect could result from defects in M4 or pharyngeal muscle development [15].

Here we explore the role of CEH-28 and ZAG-1 in regulating gene expression in M4, and we find that these factors function in a hierarchical pathway to progressively regulate distinct aspects of M4 differentiation. In addition to activating *dbl-1*, CEH-28 activates expression of the FGF gene *egl-17* and the FMRFamide peptide genes *flp-5* and *flp-2*. In contrast, ZAG-1 functions upstream and activates expression of *ceh-28* and its downstream targets, but it also is necessary for expression of *ser-7b*, which is expressed independently of CEH-28 [12]. Other genes are expressed normally in M4 in both *ceh-28* and *zag-1* mutants, indicating neither of these factors is a terminal selector of M4 fate [16]. This understanding of how these conserved factors function in M4 may guide work developing therapies by manipulating mammalian ZAG-1 and CEH-28 orthologs to produce specific neuronal differentiation patterns.

## Results

### CEH-28 activates *egl-17*, *flp-5*, and *flp-2* expression in M4

**Figure 1:**
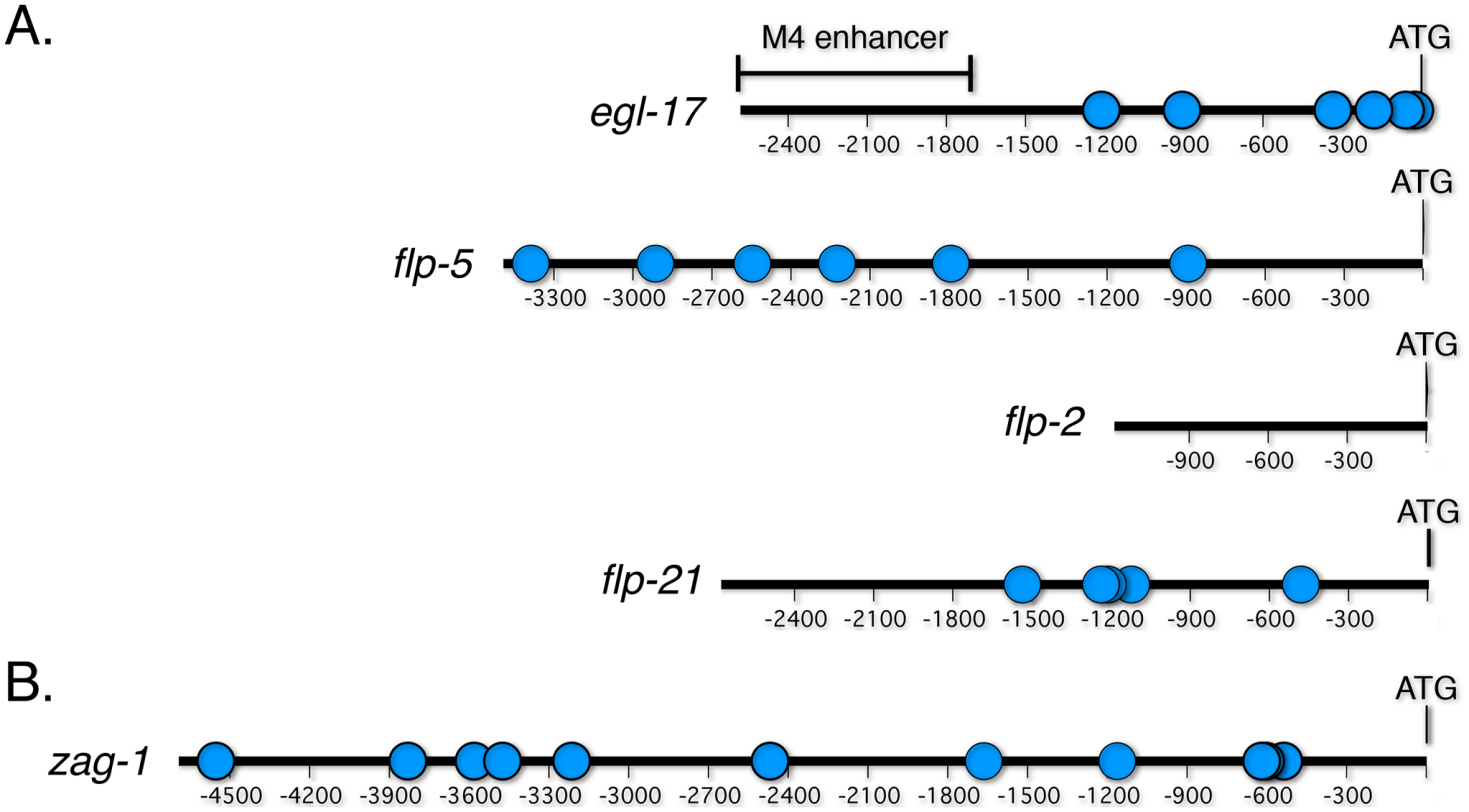
Promoters of potential CEH-28 target genes. Schematic diagrams of promoter fragments in *gfp* fusions used in this study with potential CEH-28 binding sites indicated (blue dots). The translational start site (ATG) is numbered as bp 1. (A) *egl-17* contains an M4 specific enhancer (bar). Potential CEH-28 binding sites are located in *egl-17* at −1212, −906, −334, −179, −59, and −24; in *flp-5* at −3387, −2914, −2546, −2225, −1793, and −892; in *flp-21* at −1536, −1238, −1212, −1123, and −480. (B) Schematic diagram of the *zag-1* promoter sufficient for *zag-1* expression in M4 and other neurons [15]. Our studies used fosmid WRM063aA08 containing a *gfp* translational fusion [45], which is expressed in similar pattern. *zag-1* contains potential CEH-28 binding sites at −4552, −3830, −3581, −3474, −3214, −2468, −1664, −1162, −619, −604, and −536.

CEH-28 is an NK-2 family homeodomain transcription factor that is expressed exclusively in the M4 pharyngeal neuron from mid-embryogenesis through adulthood, and it regulates M4 synapse assembly and signaling [9], [12]. The only previously known transcriptional target of CEH-28 is *dbl-1*, which encodes a TGF-ß family growth factor secreted from M4 to affect the nearby g1 pharyngeal gland cells [9]. We sought to identify additional targets by comparing expression of *gfp* reporters regulated by the *egl-17*, *flp-5*, *flp-2* and *flp-21* promoters in wild-type animals and *ceh-28* mutants (Figure 1A). These reporters are expressed in M4 [10], [11], and some contain potential CEH-28 binding sites, suggesting they may be direct targets of CEH-28 regulation.

**Figure 2:**
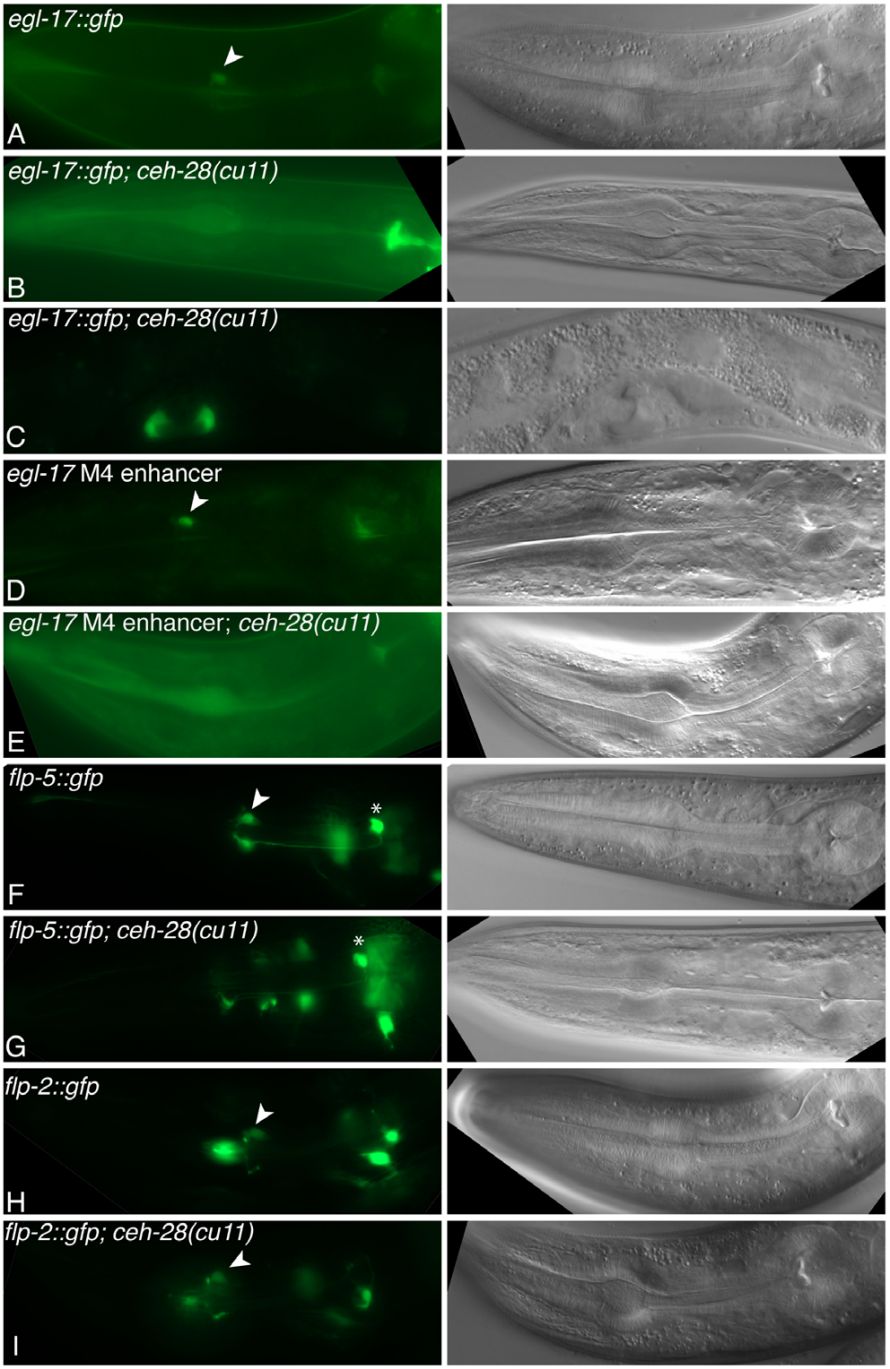
Expression of M4 differentiation markers in *ceh-28(cu11)* mutants. Fluorescence (left) and DIC (right) micrographs of L4 to adult animals of the indicated genotypes bearing *egl-17::gfp ayIs4* (A–C), the *egl-17* M4 enhancer::*Δpes-10::gfp cuEx793* (D,E), the *flp-5::gfp ynIs49* (F,G), or the *flp-2::gfp ynIs57* (H,I). (A,B,D–I) Expression in the pharynx with M4 (arrowhead) or I4 (asterisk, F and G) indicated. (C) *egl-17::gfp* expression in the vulva, which is unaffected in *ceh-28* mutants.

*egl-17* encodes a fibroblast growth factor (FGF) expressed in M4 and the vulva [10], and we found that CEH-28 activates *egl-17* expression specifically in M4. *egl-17::gfp* expression was completely lost in M4 in *ceh-28* mutants, while expression in the vulva was unaffected (Figure 2A–C; Table 1). In the *dbl-1* promoter, separable sequences mediate expression in M4 and other neurons, and CEH-28 directly targets an M4-specific enhancer in this promoter [9]. Previous studies suggest the *egl-17* promoter has a similar organization [17]. This work identified a region from −2589 to −1756 bp upstream of the translational start site necessary for *egl-17::gfp* expression in M4, but it had no role in vulval cell expression. We asked if this fragment was sufficient to enhance expression of the basal *pes-10* promoter fused to *gfp* (*Δpes-10::gfp*), which is sensitive to linked enhancers [18]. We found transgenic animals bearing this reporter expressed GFP exclusively in M4 (Figure 1A; Figure 2D). While this enhancer does not contain any recognizable CEH-28 binding sites, its activity was lost in *ceh-28* mutants, indicating that it functions downstream of CEH-28 (Figure 2E; Table 1). We suggest either that enhancer is directly activated by CEH-28 through non-consensus binding sites, or it is activated indirectly by another CEH-28 dependent factor.

**Table 1:** Frequency of animals expressing GFP in M4 in wild-type and *ceh-28* mutants.

Reporter	Percent animals expressing GFP inM4 in wild type (n)^a^	Percent animals expressing GFPin M4 in *ceh-28(cu11)* (n)^a^^,^ b
*ayIs4[egl-17::gfp]*	100 (35)	0 (40)**
*egl-17 M4 enhancer::gfp*	80 (30)	0 (30)**
*ynIs49[flp-5::gfp]*	100 (30)	0 (37)**
*ynIs57[flp-2::gfp]*	100 (30)	80 (45)*
*ynIs80[flp-21::gfp]*	100 (32)	100 (35)
*wgIs83[zag-1::gfp]*	100 (40)	66 (45)**

a Transgenic adults were scored for GFP expression in M4.

b Statistically significant difference between *ceh-28(cu11)* and wild type. (*p<0.01; **p<0.0001). Calculated using the two-tailed, Fisher’s exact test.

*flp-2*, *flp-5* and *flp-21* encode FMRFamide-like neuropeptides expressed in M4 and other neurons, and we found that CEH-28 activates *flp-5* and *flp-2* expression in M4, but *flp-21* is expressed independently of CEH-28. Expression of *flp-5::gfp* was eliminated in *ceh-28* mutant M4 cells, while the frequency of *flp-2::gfp* expression was modestly but significantly reduced (Figure 2F–I, Table 1). In both cases expression was unaffected in other neurons. In contrast, *flp-21::gfp* expression was unaffected in M4 and other neurons in *ceh-28* mutants (Table 1).

These results expand our understanding of gene regulation in M4, and together with our previous work, identify *dbl-1*, *egl-17*, and *flp-5* as downstream targets of CEH-28 [9], [12]. CEH-28 contributes to *flp-2* expression, but other factors must also activate *flp-2* in M4. In contrast *ser-7b*, *unc-17*, and *flp-21* are expressed in M4 independently of CEH-28 [12].

### ZAG-1 is essential for isthmus peristalsis

ZAG-1 is a ZEB-family C_2_H_2_ zinc-finger/homeodomain factor that regulates neuron pathfinding and differentiation in *C. elegans* [14], [15]. It is believed to be expressed in M4 and many other neurons, and in some pharyngeal muscles during embryogenesis. *zag-1(hd16)* null mutants arrest after hatching and exhibit a stuffed pharynx phenotype [15]. Because this phenotype can result from M4 defects, we characterized pharyngeal muscle contractions and M4 function in *zag-1(hd16)* mutants.

We found *zag-1(hd16)* mutants completely lack isthmus peristalses. These mutants pump, although at a slower rate than wild-type L1s (Table 2; Movie S1 and S2). However, while wild-type L1s peristalse approximately after every 9th pump, *zag-1(hd16)* mutants never exhibited a peristalsis (Table 2). Both of these phenotypes are observed in animals lacking M4 [5], [19], suggesting motor neuron function of M4 is defective in *zag-1* mutants.

**Table 2:** Summary of feeding behavior in wild-type and *zag-1* mutants.

Genotype	Pump Rate (pumps/min)	Duration of Procorpus contractions (ms)	Duration of Posterior Bulb Contractions (ms)	Duration of Isthmus peristalsis (ms)	% Pumps followed by Isthmus Peristalsis
N2^a^	116±2	159±1	173±2	n.d.	11%
*zag-1(hd16)* b	58±21	118±3	96±6	n.d.	0%
N2+ serotonin^c^	177±39	n.d.	n.d.	n.d.	30%
*zag-1(hd16)*+ serotonin^d^	158±41	n.d.	n.d.	n.d.	0%
N2+ arecoline^e^	50±16	n.d.	n.d.	216±22	100%
*zag-1(hd16)* + arecoline^f^	34±4	n.d.	n.d.	128±22	100%

a 4 N2 L1s were recorded for 35–40 s and a total of 213 pumps were analyzed.

b 4 *zag-1(hd16)* L1s were recorded for 35–60 s and a total of 203 pumps were analyzed.

c 4 N2 L1s were treated with 20 mM serotonin and recorded for 15–20 s each and a total of 193 pumps were analyzed.

d 5 *zag-1(hd16)* L1s were treated 20 mM serotonin and recorded for 12–25 s each and a total of 222 pumps were analyzed.

e 4 N2 L1s were treated with 5 mM arecoline and recorded for 28–50 s each and a total of 115 pumps were analyzed.

f 6 *zag-1(hd16)* L1s were treated with 5 mM arecoline and recorded for 45–60 s each and a total of 166 pumps were analyzed.

To determine if the lack of peristalses in *zag-1(hd16)* mutants results from defects in M4 or the pharyngeal muscles, we examined pharyngeal muscle contractions in animals treated with compounds that stimulate either of these cell types. Serotonin stimulates the MC and M4 neurons, and this leads to increased pumping and peristalsis, respectively [20]. Wild-type L1s treated with serotonin exhibited a moderate increase in the pump rate and frequency of peristalsis compared to untreated animals (Table 2; Movie S3). In comparison, *zag-1(hd16)* mutants treated with serotonin exhibited a strong increase in the pump rate compared to untreated animals, but they still failed to peristalse (Table 2; Movie S4). Arecoline directly stimulates acetylcholine receptors in the isthmus muscles [12], [19], and we found that arecoline treatment stimulated very frequent peristalses in both wild-type L1s and *zag-1(hd16)* mutants (Table 2; Movies S5 and S6). Together these results demonstrate that the isthmus muscle of *zag-1(hd16)* mutants can produce a peristaltic contraction, but the M4 cell in these animals cannot stimulate this contraction. While arecoline treated *zag-1* mutants did peristalse, these contractions were shorter than those in wild-type animals, suggesting the functional M4 in wild-type animals still affects peristalsis under these conditions (Table 2).

### ZAG-1 regulates *ceh-28* and other markers of M4 differentiation

*zag-1* mutants exhibit differentiation defects in several neurons outside of the pharynx [14], [15], and we were interested in asking if M4 differentiation is similarly affected in these mutants. To examine *gfp* reporter gene expression, *zag-1(hd16)/+* hermaphrodites that were heterozygous for these chromosomally integrated reporters were generated, and we compared reporter expression in progeny *zag-1(hd16)* homozygotes and their viable wild-type or heterozygous *zag-1(hd16)/+* siblings at the L1 stage.

**Figure 3:**
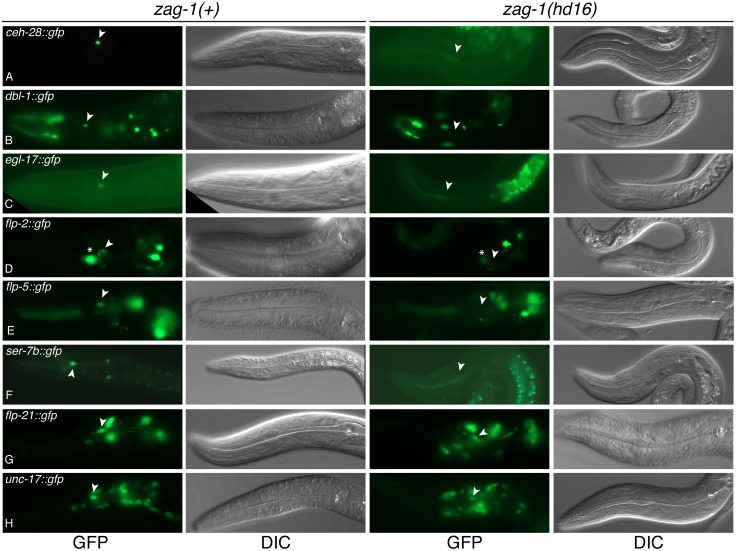
Expression of M4 differentiation markers in *zag-1(hd16)* mutants. Fluorescence (left) and DIC (right) micrographs of transgenic L1-L2 animals bearing *ceh-28::gfp* (A), *dbl-1::gfp* (B), *egl-17::gfp* (C), *flp-2::gfp* (D), *flp-5::gfp* (E), *ser-7b::gfp* (F), *flp-21::gfp* (G), *unc-17::gfp* (H) in a *zag-1(+)* or *zag-1(hd16)* mutants. The position of M4 is marked (arrowheads). Asterisk indicates *flp-2::gfp* expression in the MC neuron (D).

We first examined expression of *ceh-28::gfp* and reporters for the CEH-28 targets *dbl-1*, *egl-17*, *flp-5* and *flp-2*. Both the frequency and intensity of *ceh-28::gfp* expression was reduced in *zag-1(hd16)* homozygotes (Figure 3A; Table 3). Likewise, we also observed loss or reduced expression of the CEH-28 targets, strongly suggesting that expression of endogenous *ceh-28* is reduced in *zag-1(hd16)* homozygotes (Figure 3B–E; Table 3). The only CEH-28 target that retained some expression in *zag-1(hd16)* mutants was *flp-2::gfp*, which was also only partially affected in *ceh-28* mutants (Table 1).

**Table 3:** Expression of M4 differentiation markers in *zag-1(+)* and *zag-1(hd16)* mutants.

Transgene	% animals expressing GFP in M4 in *zag-1(+)* (n)^a^	% animals expressing GFP in M4 in *zag-1(hd16)* (n)^b^^,^ c
*nIs177[ceh-28::gfp]* d	69 (54)	22 (58)**
*ctIs43[dbl-1::gfp]* d	74 (43)	0 (32)**
*ayIs4[egl-17::gfp]* d	74 (34)	0 (36)**
*ynIs49[flp-5::gfp]* d	63 (40)	0 (40)**
*ynIs57[flp-2::gfp]* d	67 (30)	38 (45)*
*cuEx469[ser-7b::gfp]* e	63 (30)	0 (30)**
*ynIs80[flp-21::gfp]* d	74 (34)	68 (25)
*mdIs18[unc-17::gfp]* d	65 (40)	73 (30)

a GFP expression in the phenotypically wild-type *+/+* or *zag-1(hd16)/+* progeny of *zag-1(hd16)/+* hermaphrodites, which we refer to as *zag-1(+)*.

b GFP expression in the *zag-1(hd16)* homozygous progeny of *zag-1(hd16)/+* hermaphrodites.

c Statistically significant difference between *zag-1(hd16)* and *zag-1(+)*. (*p<0.02; **p<0.0001). Calculated using the two-tailed, Fisher’s exact test.

d Chromosomally integrated transgene is expected to be present in 75% of the progeny of transgenic hermaphrodites.

e *cuEx469* is an extrachromosomal transgene that is unlinked to any of the chromosomes.

We next examined reporters for the *ser-7b*, *flp-21*, and *unc-17* genes that are expressed in M4 independently of CEH-28 (Table 1) [12]. While *flp-21::gfp* and *unc-17::gfp* were expressed normally in *zag-1(hd16)* mutants, expression of *ser-7b::gfp* was completely lost (Figure 3F–H; Table 3). This loss is consistent with our observation that *zag-1(hd16)* mutants do not peristalse when treated with serotonin, and it suggests that ZAG-1 is essential for endogenous *ser-7b* expression in M4.

### CEH-28 activates *zag-1* expression in a positive feedback loop

**Figure 4:**
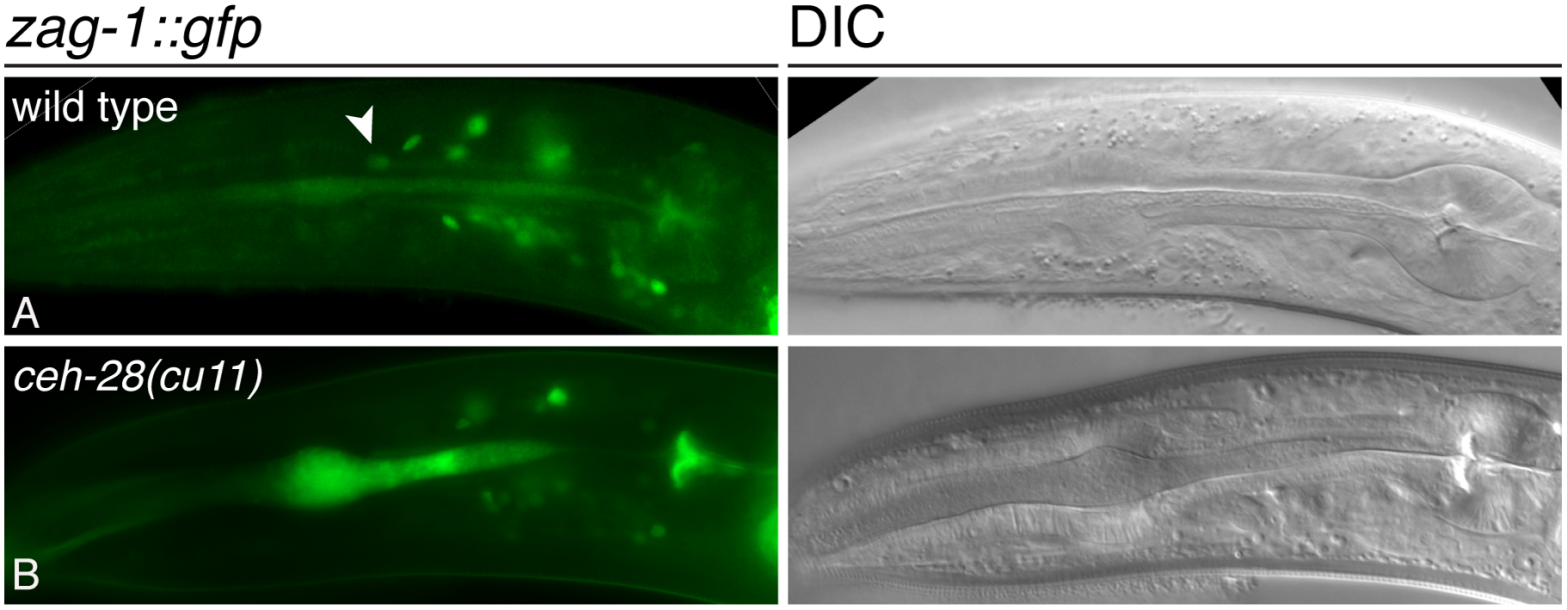
Expression of *zag-1::gfp* in wild-type and *ceh-28* mutants. Fluorescence (left) and DIC (right) micrographs of *zag-1::gfp wgIs83* in wild-type (A) and. *ceh-28(cu11)* mutant (B) adults. M4 is indicated (arrowhead) in (A). The anterior pharyngeal lumen is stuffed with bacteria in (B), and this results in non-specific autofluorescence.

While ZAG-1 functions upstream to activate expression of *ceh-28*, we observed that the *zag-1* promoter also contains several potential CEH-28 binding sites (Figure 1B). To test if CEH-28 also regulates *zag-1* expression, we examined expression of a *zag-1::gfp* reporter and found that it is indeed expressed in M4 as previously suggested (Figure 4A) [15]. The frequency of *zag-1::gfp* expression was moderately but significantly reduced in *ceh-28(cu11)* mutants compared to wild type (Figure 4B; Table 1), indicating CEH-28 functions in a positive feedback loop to activate *zag-1* expression, perhaps to maintain stable expression of both *zag-1* and *ceh-28* after initial activation.

## Discussion

**Figure 5:**
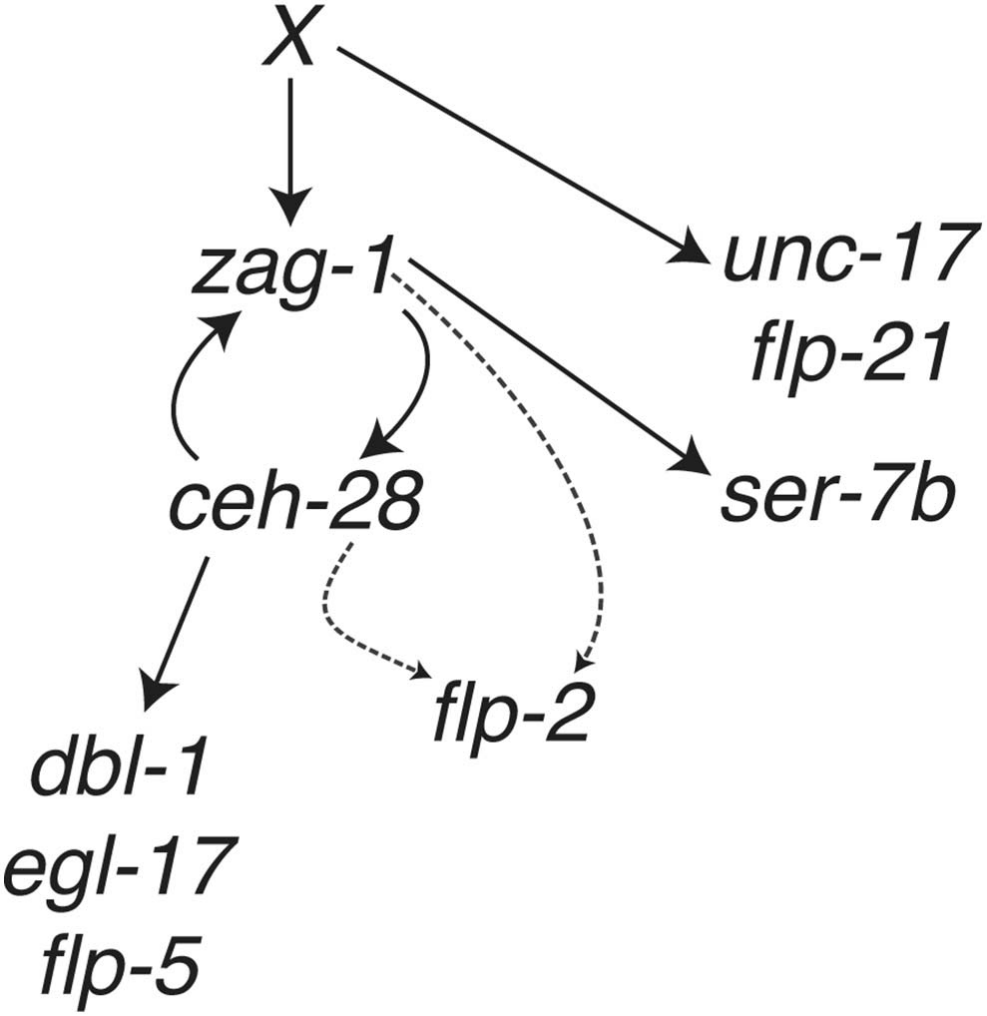
*zag-1* and *ceh-28* function in a hierarchy in the M4 neuron. Model indicating gene regulatory interactions in M4 discussed in the text. CEH-28 is necessary to activate expression of *dbl-1*, *egl-17*, and *flp-5*. CEH-28 also activates *zag-1* gene expression in a positive feedback loop. Either CEH-28, ZAG-1 or both activate *flp-2* expression (dashed arrows). ZAG-1 functions upstream to activate *ceh-28* and *ser-7b* expression, while another factor(s) activate *zag-1*, *unc-17*, and *flp-21* (indicated as X).

Here we show that the transcription factors CEH-28 and ZAG-1 function in a hierarchy to regulate multiple aspects of M4 differentiation (Figure 5). We previously showed that *ceh-28* mutants fail to express the TGF-ß family gene *dbl-1* in M4 [9], and we now find these mutants lack or exhibit reduced expression of a subset of additional M4 differentiation markers, including *egl-17::gfp*, *flp-5::gfp* and *flp-2::gfp*. We also find that ZAG-1 functions upstream of CEH-28 and plays a broader role in M4 differentiation. *zag-1* mutants exhibit strongly reduced frequency and intensity of expression of a *ceh-28::gfp* marker, and reduced or eliminated expression of markers regulated by CEH-28. We hypothesize that ZAG-1 is required for strong expression of the endogenous *ceh-28* gene in M4, and the reduced *ceh-28* expression in *zag-1* mutants leads to loss of expression of the CEH-28 downstream targets *dbl-1*, *egl-17* and *flp-5*. Notably, *zag-1* mutants also lack expression of a *ser-7b::gfp*, which is expressed normally in *ceh-28* mutants, demonstrating ZAG-1 functions upstream of CEH-28 and regulates the *ser-7* promoter independently of CEH-28 [12]. *zag-1::gfp* expression is also reduced in *ceh-28* mutants, indicating CEH-28 contributes to *zag-1* expression through a positive feedback loop. While *flp-2::gfp* expression is reduced in both *zag-1* and *ceh-28* mutants, it is difficult to know if *flp-2* is directly downstream of either of these genes, or whether both function in parallel to activate *flp-2* (Figure 5). Because of positive feedback between *ceh-28* and *zag-1*, mutations affecting in either of these alter expression of the other gene, which in turn could *flp-2* expression. Finally, the M4 differentiation markers *unc-17::gfp* and *flp-21::gfp* are expressed normally in both *zag-1* and *ceh-28* mutants, indicating other factors promote aspects of M4 neuronal differentiation independently of ZAG-1 and CEH-28. We suggest an additional factor(s) (‘X’ in Figure 5) activates expression of these genes, as well as *zag-1* in M4.

We further show that defects in M4 differentiation in *zag-1* mutants result in an absence of peristaltic contractions in the pharyngeal isthmus muscles. This phenotype results from defects in the M4 neuron rather than in the muscle, because stimulating the isthmus muscles with arecoline stimulates isthmus peristalsis in *zag-1* mutants, whereas stimulating M4 with serotonin has no effect in these animals. This severe peristalsis defect likely contributes to the L1 arrest phenotype of *zag-1(hd16)* as previously suggested [15].

### CEH-28 regulates M4 signaling by activating growth factors and neuropeptides

CEH-28 is not generally required for M4 neuronal differentiation. However, *ceh-28* mutants are defective in expressing *dbl-1*, *egl-17*, *flp-5*, and *flp-2* [9], [12], indicating that CEH-28 regulates multiple neurosecretory functions of M4. Like *dbl-1*, the *egl-17, flp-5* and *flp-2* genes are also expressed in cells other than M4, and this expression is unaffected in *ceh-28* mutants. Both *dbl-1* and *egl-17* contain M4 specific enhancers that are separable from sequences controlling expression in other cells [9], [17], and this modular organization may be a common feature of promoters active in M4. Activity of the *dbl-1* enhancer depends on CEH-28 binding sites [9], but, while the *egl-17* promoter region contains potential CEH-28 sites, none of these are located in the M4 enhancer (Figure 1). We speculate that this enhancer is activated by CEH-28 through non-consensus sites or through other CEH-28-dependent factors. Additional studies are necessary to determine the functional significance of potential binding sites in other promoters regulated by CEH-28.

DBL-1 secreted from M4 affects the morphology of the nearby pharyngeal g1 gland cells [9], but the functions of the newly identified CEH-28 targets in M4 are unknown. EGL-17 has no known role in the pharynx, while exogenous FLP-5 and FLP-2 neuropeptides can excite pumping in pharyngeal explants [21]. None of the mutants *egl-17(n1377), flp-5(gk3123)* or *flp-2(gk1039)* exhibit a stuffed pharynx phenotype similar to that of *ceh-28* mutants, suggesting these secreted proteins are not necessary for normal feeding (data not shown), and we believe other CEH-28 targets are important for M4 synapse assembly and motor neuron function. Alternatively, the functions of these genes are redundant with each other or with other signaling pathways, as has been observed for cholinergic and neuropeptide control of egg laying [22].

### ZAG-1 plays a crucial role in regulating M4 differentiation

ZAG-1 is an ortholog of the vertebrate ZEB family transcription factors and Drosophila Zfh1 [14], [15]. In vertebrates these proteins regulate epithelial to mesenchymal transitions during development and in cancer metastasis, and control differentiation of particular neuronal types [13], [23]. Mutations affecting human ZEB proteins have been implicated in Mowat Wilson syndrome and corneal dystrophies [24]–[27]. In *C. elegans* and Drosophila, ZEB family proteins function in axonal path finding, neuronal differentiation, and neuronal cell fate [14], [15], [28], [29].

Our results indicate ZAG-1 is a major regulator of M4 differentiation. M4 is present and partially differentiated in *zag-1* mutants, but these mutants lack expression of several markers of M4 differentiation. Moreover *zag-1* mutants exhibit a complete loss of peristaltic contraction of the isthmus muscles. This contractile defect results from defects in M4 rather than the pharyngeal muscles themselves, because stimulation of the muscles with exogenous arecoline restores peristalses, while stimulation of M4 with serotonin has no effect. In wild-type animals the ability of serotonin to stimulate pharyngeal pumping and peristalses is mediated by the SER-7 receptor in the MC and M4 motor neurons, respectively [20], and the failure of exogenous serotonin to simulate peristalsis in *zag-1* mutants is consistent with the loss of expression of the endogenous *ser-7* gene in M4 in these animals.

ZEB family proteins most often function as transcriptional repressors, but they can also activate transcription [reviewed in [30]]. Mammalian ZEB1 activates transcription of the ovalbumin gene in response to estrogen signaling [31], as well as the MMP-1 and CDK-4 genes [32], [33]. Likewise, Drosophila *Zfh1* can repress expression of *mef2* during muscle development [34], while it activates expression of FMRFa gene in neurons [35]. This ability of ZEB family factors to function either as activators and repressors may result from cell type specific cofactors or post-translational modifications [36]–[38] or different DNA binding activities mediated through the multiple binding domains in these proteins [39].

Like its vertebrate and Drosophila orthologs, *C. elegans* ZAG-1 also functions as both a repressor and an activator. ZAG-1 negatively regulates its own expression and expression of *unc-25*, which is required for GABA synthesis [14], [15]. Our results now suggest ZAG-1 can also function as a transcriptional activator of the *ser-7b* and *ceh-28* promoters (Figure 5). Current whole animal ChIP-seq analyses performed by the modENCODE consortium have not revealed significant ZAG-1 binding within these promoters [40], so we do not know if this regulation is direct, but binding might be undetectable if it only occurs in M4 or a small number of other cells.

Recently, ZEB2 was found to repress *Nkx-2.1* expression in the developing mouse cerebral cortex, and loss of this regulation may contribute to Mowat Wilson syndrome [23], [41], [42]. While this regulation is opposite to what we have observed between ZAG-1 and *ceh-28*, it suggests ZEB-family factors may be common regulators of NK-2 family homeobox genes.

### A hierarchy of transcription factors control M4 differentiation

In both invertebrates and vertebrates, ‘terminal selector’ transcription factors have been shown to be key activators of batteries of genes involved in terminal differentiation of specific neuronal types [reviewed in [16]]. Such terminal selector genes are expressed in these specific neurons throughout development, and, after initial activation, they maintain their own expression through positive autoregulation. While mutants defective for terminal selector genes express markers of pan-neural differentiation, they fail to express markers of neuron type-specific differentiation.

While both *ceh-28* and *zag-1* are expressed in M4 throughout development, neither appears to have other characteristics of a terminal selector for the M4 phenotype. CEH-28 does not maintain its own expression in M4, and *ceh-28* mutants strongly express a *ceh-28::gfp* reporter in M4 throughout their life-cycle [12]. In comparison, ZAG-1 does partially activate its own expression indirectly in M4 via CEH-28 in a positive feedback loop (Figure 5), but it represses its own promoter in many neurons [14], [15]. More importantly, neither of these factors appears necessary for expression of batteries of genes for a specific aspect of M4 differentiation. For example, the *flp-5, flp-2*, and *flp-21* genes encoding FMRFamide-family neuropeptides all are regulated differently in M4. Instead, our observations indicate ZAG-1 and CEH-28 function in a branched, hierarchical network to regulate M4 gene expression (Figure 5). Other genes are regulated independently of both CEH-28 and ZAG-1, and additional transcription factors must function upstream in this hierarchy. *zag-1* and *ceh-28* could themselves be activated by a terminal selector of M4 differentiation. Alternatively, different aspects of M4 differentiation might be independently regulated without a terminal selector transcription factor, perhaps resulting from the multifunctional nature of M4. More comprehensive analyses of M4 gene expression and the promoters of M4 expressed genes will distinguish between these possibilities.

## Materials and Methods

### Nematode handling, transformation and strains

*C. elegans* strains were grown under standard conditions [43]. Germline transformations were performed using pRF4 (100 ng/µl) and indicated *gfp* reporters (15 ng/µl) [44].

The following strains were used in this study: NH2466 *ayIs4[egl-17::gfp dpy-20(+)]; dpy-20(e1282ts)* [10], OK0978 *ayIs4; ceh-28(cu11)*, OK0975 *cuEx793 [egl-17 M4 enhancer]*, OK0976 *ceh-28(cu11); cuEx793*, NY2049 *ynIs49[flp-5::gfp]* [11], OK0979 *ynIs49; ceh-28(cu11)*, NY2057 *nyIs47; him-5(e1490)* [11], OK0980 *ynIs57; ceh-28(cu11)*, NY2080 *ynIs80[flp-21::gfp]* [11], OK1013 *ynIs80; ceh-28(cu11)*, OP83 *unc-119(ed3); wgIs83[zag-1::TY1::gfp::3xFLAG + unc-119(+)]* [45], OK0974 *unc-119(ed3); ceh-28(cu11); wgIs83*, VH514 *zag-1(hd16)/unc-17(ed113) dpy-14(e184)* [15] MT15672 *nIs177[ceh-28::4xNLS::gfp]* [46], BW1946 *ctIs43[Pdbl-1::gfp]; unc-42(e270)* [47], OK516 *cuEx469[ser-7b::gfp]* [12], RM2258 *pha-1(e2132ts); mdIs18[unc-17::gfp]* [48].

To visualize the expression of *gfp* reporters in *zag-1(hd16)* homozygotes, transheterozygous *zag-1(hd16)/+; gfp/+* were generated by crossing *gfp/+* males with *zag-1(hd16)/unc-17(ed113) dpy-14(e184)* hermaphrodites and picking GFP expressing F_1_ cross progeny to individual plates. F_1_ animals were allowed to produce progeny for 2 days (25°C), and *zag-1(hd16)/+; gfp/+* plates segregating *zag-1(hd16)* homozygotes in the F_2_ were identified. GFP expression was examined in F_2_ *zag-1(hd16)* homozygous progeny, recognized by their Unc, Coiler phenotype, and their +/+ or *zag-1(hd16)/+* siblings.

### General methods for nucleic acid manipulations and plasmid construction

Standard methods were used to manipulate all DNAs [49], and plasmids sequences are available from the authors. The *egl-17* M4 enhancer containing bp 18,928–19,857 of cosmid F38G1 (accession # FO080171) was amplified from N2 genomic DNA and inserted into HindIII-SalI digested *Δpes-10::gfp* to generate pOK293.01.

### Identification of candidate CEH-28 binding sites potential targets

Candidate CEH-28 binding sites were identified as described previously [9] by scanning the promoter sequences using the WormBase function Annotate Sequence Motif using the GBrowse plugin MotifFinder (www.wormbase.org;gmod.org/wiki/MotifFinder.pm) with the JASPAR position-frequency-matrix MA0264.1 (jaspar.cgb.ki.se) for the closely related homeodomain factor CEH-22 at a threshold of 0.82 [50].

### Analysis of feeding behavior and drug studies

To analyze pharyngeal muscle contractions, wild-type or *zag-1(hd16)* embryos were hatched in the absence of food on unseeded NGM plates. L1 larvae from each genotype were suspended in 5 µl of M9 buffer containing OP50 and imaged on a 2% agarose pad under a coverslip. To stimulate M4 with serotonin, L1 animals obtained from a mixed stage population were placed on an unseeded NGM plate for 20 min and subsequently soaked 20 min in 20 mM serotonin on a 2% agarose pad before imaging. Pharyngeal muscles were stimulated with 5 mM arecoline as previously described [12].

Individual N2 or *zag-1(hd16)* animals that pumped were recorded at 25 frames/sec for 2 min using a Zeiss AxioImager microscope with an MRm camera and ZEN Software. For each genotype or drug treatment the feeding behavior was analyzed in at least 4 animals. Video frames and QuickTime movies of feeding behavior were exported and processed using ImageJ (developed at the US NIH and available at http://rsb.info.nih.gov/nih-image/) and quantifications were performed using Microsoft Excel.

## Supporting Information

Movie S1 **Pumping and peristalsis in a wild-type L1 larva.** Five pumps of a wild-type L1 animal played at 1/5^th^ speed (5 frames/sec). A pump occurs when the muscles in the anterior and posterior pharynx synchronously contract to open the pharyngeal lumen. A wave-like, peristaltic contraction is observed in the isthmus only after the third pump.
(MOV)

Movie S2 ***zag-1*** **mutants completely lack isthmus peristalsis.** Seven pumps of a *zag-1(hd16)* mutant animal played at 1/5^th^ speed (5 frames/sec). Note that the animal pumps somewhat more slowly than a wild-type animal, and that peristaltic contraction in the isthmus was never observed.
(MOV)

Movie S3 **Pumping and peristalsis in serotonin treated wild-type L1 larva.** Three pumps of a wild-type L1 treated with 20 mM serotonin played at 1/5^th^ speed (5 frames/sec). A peristaltic contraction was observed only after the second pump.
(MOV)

Movie S4 **Feeding behavior of serotonin treated** ***zag-1(hd16*** **) mutants.** Seven pumps of a *zag-1(hd16)* mutant L1 larva treated with 20 mM serotonin played at 1/5^th^ speed (5 frames/sec). Note that the animal pumps normally, however a peristaltic contraction in the isthmus.
(MOV)

Movie S5 **Wild-type L1 larva treated with acetylcholine receptor agonist arecoline.** Four pumps of the wild-type L1 treated with 5 mM arecoline played at 1/5^th^ speed (5 frames/sec). Note that every pump is followed by a prolonged peristaltic contraction in which a larger region of the isthmus lumen is open at any given time.
(MOV)

Movie S6 ***zag-1(hd16)*** **mutant L1 larva treated with acetylcholine receptor agonist arecoline.** Two pumps of a *zag-1(hd16)* mutant L1 treated with 5 mM arecoline played at 1/5^th^ speed (5 frames/sec). Both the pumps are followed by a strong peristaltic contraction.
(MOV)
